# The Protective Effect of Phaseolus Vulgaris on Cataract in Type 2 Diabetes: A Profitable Hypothesis

**Published:** 2013

**Authors:** Benjamin Longo-Mbenza, MoiseMvitu Muaka

**Affiliations:** 1 Walter Sisulu University, Faculty of Health Sciences, South Africa,; 2 University of Kinshasa, Faculty of Medicine Service of Ophthalmology, Kinshasa, Democratic Republic of the Congo

**Keywords:** *Phaseolus vulgaris*, Visual deficiencies, Antioxidant, Cataract, Diabetes Mellitus, Anti-radical Activity

## Abstract

The pathophysiology of major ocular complications in type 2 diabetes mellitus (T2DM) among Bantu is not well understood. Several studies have been conducted to determine the basic reasons of visual deficiencies (VD) (blindness, visual impairment, and ocular eye diseases) in T2DM among Bantu from Central Africa. The quality of dietary intake was assessed in patients along with other ophthalmological assessments for diabetic retinopathy, cataract, glaucoma, and macular edema. Beans (Phaseolus vulgaris) and leafy vegetables are rich in antioxidants. The consumption of at least 3 ladles per meal, 3 times or more per week, has been identified as a potential protective factor against cataract. The anti-radical activity of beans is well known in the literature. Beans are considered to have a comparatively higher antioxidant activity than in many other vegetables. Our findings from previous epidemiologic studies establish that the antioxidant activity of P. vulgaris helps control blood glucose. We, therefore, hypothesize that the dietary supplements of bean can be a low-cost prevention approach to reduce cataract and much other visual comorbidity associated with T2DM. However, further epidemiological studies combined with molecular research need to be conducted to prove this hypothesis.

## INTRODUCTION

Type 2 diabetes mellitus (T2DM) is a lifelong disease, which is characterized by high levels of glucose in the blood. Worryingly, it is growing at an alarming rate in sub-saharan Africa. From the literature, it has been elucidated that the major risk factors are westernization, sedentary lifestyle, metabolic syndrome (MetS), and nutritional transition contributing to overweight (1-3). The leading causes of blindness in diabetic patients comprise diabetic retinopathy (DR), cataract, glaucoma, macular edema, age-related macular degeneration, (AMD) and optic nerve atrophy. T2DM is also associated with high incidence of risk factors for developing cardiovascular disease (CVD) (4-11). An eye survey showed that in the US, almost 3.4 million of adults aged 40 or more were visually disabled and main causes were DR and age-related eye diseases (cataract, AMD, and glaucoma) (12). Likewise, DR, cataract, glaucoma, and AMD also appear to be the major causes of blindness in ophthalmic practice in Kinshasa, Matadi, Boma, Muanda, and Mayombe (MvituMuaka, Unpublished Data). We reported 12% cases of DM in Central Africa, which was more than that reported in the prior published manuscripts. This finding clearly indicates that the prevalence of diabetes in this part of world is increased. The study showed that the three main causes of blindness accounting for 47%, 33%, and 19.8% of cases were DR, cataract, and glaucoma, respectively (13).

 Ample evidence is available confirming that oxidative stress increases in patients with T2DM (14,15). Several studies in the literature support that the antioxidant rich diet-the diet based on beans and vegetables—prevents cataract formation by inhibiting the oxidation of lipids and proteins in the crystalline lens (16,17). 

**Figure 1:**
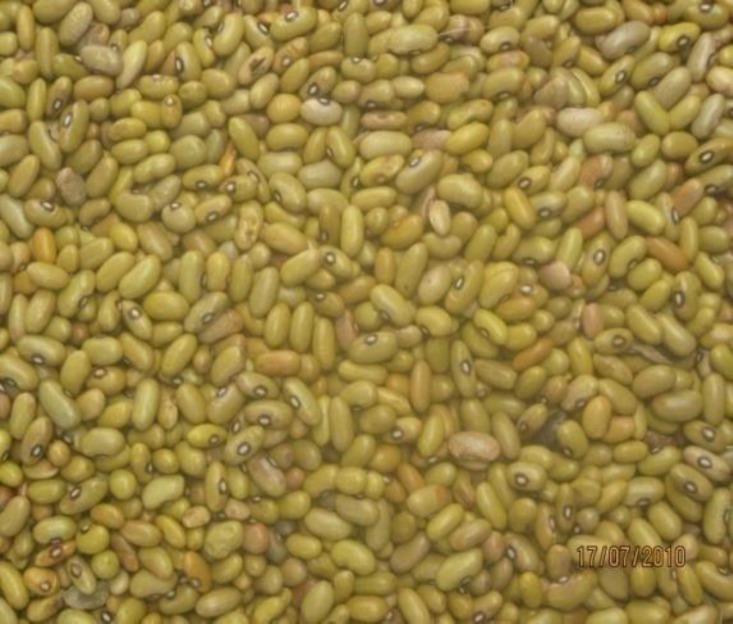
Phaseolus vulgaris (Bean)

 P. vulgaris or bean (Figure 1) is a mass-consumed food in Central Africa, and its anti-radical activity, blood glucose control property, and weight loss property have been substantiated by a growing body of evidence (18-26). The nutritional composition of bean includes the following: trivial fat and an insignificant amount of saturated fat, phytate and phenolic compositions (which act in the same way as glucose-lowering alpha-glucosidase or alpha-amylase inhibitors, e.g., metformin and acarbose), high folate, Fe, Mg, Zn, omega-3 fatty acids, and antioxidants (27-32). It has also been elucidated that the antioxidant content of red beans is the highest among vegetables (33).

## HYPOTHESES

Considering the evidence which has been discussed, we hypothesize that beans can be a cheap and healthy dietary supplement to effectively prevent or at least retard the progress of cataract and visual comorbidities associated with T2DM in diabetic patients. 

## DISCUSSION

Among the native populations of Congo, the consumption of red beans was considered as a preventive measure for metabolic syndrome (34) and cataract (35) and other visual impairments. We conducted a cross-sectional study in Congo using 244 patients with T2DM. This was the first African study on the prevention of cataract that involved the consumption of vegetables rich in antioxidants, in general, and red beans, specifically. The result showed an independent and protective role of dietary antioxidant-red beans and vegetables-against cataract development, and only red beans were independent and significantly possible protective factors against cataract surgery. Therefore we suggested the intake of antioxidant-rich vegetables, including red beans (35). 

 In another cross-sectional study conducted among 500 type 2 diabetic patients in Kinshasa, Africa, we found that with regular intake of P. vulgaris (beans), the absolute risk of cataract significantly reduced (36). 

 Likewise, a majority of literature suggest that the regular intake of antioxidants, e.g., red beans and vegetables, precludes cataract development as these antioxidants inhibit the oxidation of proteins and lipids oxidation (37-39) in the lens. 

## CONCLUSION

In conclusion, P. vulgaris might be a cost-effective dietary supplement to prevent or stop cataract formation in diabetic patients because of its well-known antioxidant activity, especially its blood glucose control property. However, further good quality designed epidemiological studies combined with molecular research are required to explore the molecular details of these properties, which may open new horizons with less side effects and more cost-effective pharmacologic management of cataract.

## References

[1] Longo-Mbenza B, Ngoma DV, Nahimana D, Mayuku DM, Fuele SM, Ekwanzala F, Beya C (2008). Screen detection and the WHO stepwise approach to the prevalence and risk factors of arterial hypertension in Kinshasa. Eur J Cardiovasc Prev Rehabil.

[2] On'Kin JB, Longo-Mbenza B, Okwe N, Kabangu NK, MpandamadiSD (2008). Prevalence and risk factors of diabetes mellitus in Kinshasa Hinterland. Int J Diabetes Metabolism.

[3] Longo-Mbenza B, On'kin JB, Okwe AN, Kabangu NK, Fuele SM (2010). Metabolic syndrome, aging, physical inactivity, and incidence of type 2 diabetes in general African population. Diab Vasc Dis Res.

[4] Al-Akily SA, Bamashmus MA, Gunaid AA (2011). Causes of visual impairment and blindness among Yemenis with diabetes: a hospital-based study. East Mediterr Health J.

[5] KasiamLasiOn'kin JB, Longo-Mbenza B, NgeOkwe A, KangolaKabangu N (2007). Survey of abdominal obesities in an adult urban population of Kinshasa, Democratic Republic of Congo. Cardiovasc J Afr.

[6] Longo-Mbenza B, Ngoma DV, Nahimana D, Mayuku DM, Fuele SM, Ekwanzala F, Beya C (2008). Screen detection and the WHO stepwise approach to the prevalence and risk factors of arterial hypertension in Kinshasa. Eur J Cardiovasc Prev Rehabil.

[7] Al-Till MI, Al-Bdour MD, Ajlouni KM (2005). Prevalence of blindness and visual impairment among Jordanian diabetics. Eur J Ophthalmol.

[8] Misra A, Khurana L (2008). Obesity and the metabolic syndrome in developing countries. J Clin Endocrinol Metab.

[9] Schémann JF, Inocencio F, de Lourdes Monteiro M, Andrade J, Auzemery A, Guelfi Y (2006). Blindness and low vision in Cape Verde Islands: results of a national eye survey. Ophthalmic Epidemiol.

[10] Hong Y (2009). Burden of cardiovascular disease in Asia: big challenges and ample opportunities for action and making a difference. Clin Chem.

[11] NACB LMPG Committee Members, Myers GL, Christenson RH, Cushman M, Ballantyne CM, Cooper GR, Pfeiffer CM, Grundy SM, Labarthe DR, Levy D, Rifai N, Wilson PW (2009). National Academy of Clinical Biochemistry Laboratory Medicine Practice guidelines: emerging biomarkers for primary prevention of cardiovascular disease. Clin Chem.

[12] Centers for Disease Control, Prevention (CDC) (2004). Prevalence of visual impairment and selected eye diseases among persons aged >/=50 years with and without diabetes--United States, 2002. MMWR Morb Mortal Wkly Rep.

[13] MvituMuaka M, Longo-Mbenza B, KaimboWaKaimbo D (2009). Frequency and causes of blindness and poor vision in Congolese diabetic patients. Mali Med.

[14] McColl AJ, Kong C, Nimmo L, Collins J, Elkeles RS, Richmond W (1997). Total antioxidant status, protein glycation, lipid hydroperoxides in non insulin dependent diabetes mellitus. Biochem Soc Trans.

[15] Venkateswaran S, Pari L (2002). Antioxidant effect of Phaseolus vulgaris in streptozotocin-induced diabetic rats. Asia Pac J ClinNutr.

[16] Rumley AG, Paterson JR (1998). Analytical aspects of antioxidants and free radical activity in clinical biochemistry. Ann Clin Biochem.

[17] Thiagarajan R, Manikandan R (2013). Antioxidants and cataract. Free Radic Res.

[18] Venkateswaran S, Pari L (2002). Antioxidant effect of Phaseolus vulgaris in streptozotocin-induced diabetic rats. Asia Pac J Clin Nutr.

[19] Dragsted LO, Krath B, Ravn-Haren G, Vogel UB, Vinggaard AM, Bo Jensen P, Loft S, Rasmussen SE, Sandstrom Tl, Pedersen A (2006). Biological effects of fruit and vegetables. Proc Nutr Soc.

[20] Layer P, Zinsmeister AR, DiMagno EP (1986). Effects of decreasing intraluminal amylase activity on starch digestion and postprandial gastrointestinal function in humans. Gastroenterology.

[21] Boivin M, Flourie B, Rizza RA, Go VL, DiMagno EP (1988). Gastrointestinal and metabolic effects of amylase inhibition in diabetics. Gastroenterology.

[22] Celleno L, Tolaini MV, D'Amore A, Perricone NV, Preuss HG (2007). A Dietary supplement containing standardized Phaseolus vulgaris extract influences body composition of overweight men and women. Int J Med Sci.

[23] Wu X, Xu X, Shen J, Perricone N, Preuss H (2010). Enhanced weight loss from a dietary supplement containing standardized Phaseolus vulgaris extract in overweight men and women. Journal of Applied Research.

[24] Thom E (2000). A randomized, double-blind, placebo-controlled trial of a new weight-reducing agent of natural origin. J Int Med Res.

[25] Koike T, Koizumi Y, Tang L, Takahara K, Saitou Y (2005). The antiobesity effect and the safety of taking “Phaseolamin(TM) 1600 diet”. J New Rem &Clin.

[26] Udani J, Hardy M, Madsen DC (2004). Blocking carbohydrate absorption and weight loss: a clinical trial using Phase 2 brand proprietary fractionated white bean extract. Altern Med Rev.

[27] Sievenpiper JL, Kendall CW, Esfahani A, Wong JM, Carleton AJ, Jiang HY, Bazinet RP, Vidgen E, Jenkins DJ (2009). Effect of non-oil-seed pulses on glycaemic control: a systematic review and meta-analysis of randomised controlled experimental trials in people with and without diabetes. Diabetologia.

[28] Aykroyd WR, Doughty J, Walker AF (1982). Legumes in Human Nutrition.

[29] Halvorsen BL, Holte K, Myhrstad MC, Barikmo I, Hvattum E, Remberg SF, Wold AB, Haffner K, Baugerød H, Andersen LF, Moskaug Ø, Jacobs DR Jr, Blomhoff R (2002). A systematic screening of total antioxidants in dietary plants. J Nutr.

[30] Kalogeropoulos N, Chiou A, Ioannou M, Karathanos VT, Hassapidou M, Andrikopoulos NK (2010). Nutritional evaluation and bioactive microconstituents (phytosterols, tocopherols, polyphenols, triterpenic acids) in cooked dry legumes usually consumed in Mediterranean countries. Food Chemistry.

[31] Lucier G, Jerardo A (2006). Vegetables and Melons Outlook. Electronic Outlook Report from the Economic Research Service no.VGS-317.

[32] Mitchell DC, Lawrence FR, Hartman TJ, Curran JM (2009). Consumption of dry beans, peas, and lentils could improve diet quality in the US population. J Am Diet Assoc.

[33] Rumawas ME, Meigs JB, Dwyer JT, McKeown NM, Jacques PF (2009). Mediterranean-style dietary pattern, reduced risk of metabolic syndrome traits, and incidence in the Framingham Offspring Cohort. Am J Clin Nutr.

[34] Muaka MM, Longo-Mbenza B, Mona DT, Okwe AN (2010). Reduced risk of metabolic syndrome due to regular intake of vegetables rich in antioxidants among African type 2 diabetics. Diabetes & Metabolic Syndrome: Clinical Research & Reviews.

[35] Mvitu M, Longo-Mbenza B, Tulomba D, Nge A (2012). Regular, high, and moderate intake of vegetables rich in antioxidants may reduce cataract risk in Central African type 2 diabetics. Int J Gen Med.

[36] Moïse MM, Benjamin LM, Doris TM, Dalida KN, Augustin NO (2012). Role of Mediterranean diet, tropical vegetables rich in antioxidants, and sunlight exposure in blindness, cataract and glaucoma among African type 2 diabetics. Int J Ophthalmol.

[37] Bhuyan KC, Bhuyan DK (1984). Molecular mechanism of cataractogenesis: III Toxic metabolites of oxygen as initiators of lipid peroxidation and cataract. Curr Eye Res.

[38] Pollack A, Oren P, Stark AH, Eisner Z, Nyska A, Madar Z (1999). Cataract development in sand and galactosemic rats fed a natural tomato extract. J Agric Food Chem.

[39] Bonnefoy M, Drai J, Kostka T (2002). [Antioxidants to slow aging, facts and perspectives]. Presse Med.

